# Does this patient have jugular venous distension? Vein finder‐enhanced assessment of jugular venous pressure

**DOI:** 10.1002/ccr3.3747

**Published:** 2021-01-08

**Authors:** Dean Bricker

**Affiliations:** ^1^ Department of Internal Medicine Wright State University Dayton OH USA

**Keywords:** jugular venous distention, jugular venous pressure, vein finder

## Abstract

Assessing jugular venous pressure (JVP) may be challenging yet useful for establishing the bedside diagnosis of congestive heart failure. Vein finder lights illuminate superficial veins and thus can improve visualization of the external jugular vein.

## Graphical abstract

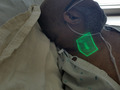

## 1 CASE

Assessing jugular venous pressure (JVP) may be challenging yet useful for establishing the bedside diagnosis of congestive heart failure. We used a vein finder light to improve visualization of the external jugular veins and enhance the assessment of JVP.

**FIGURE 1:**
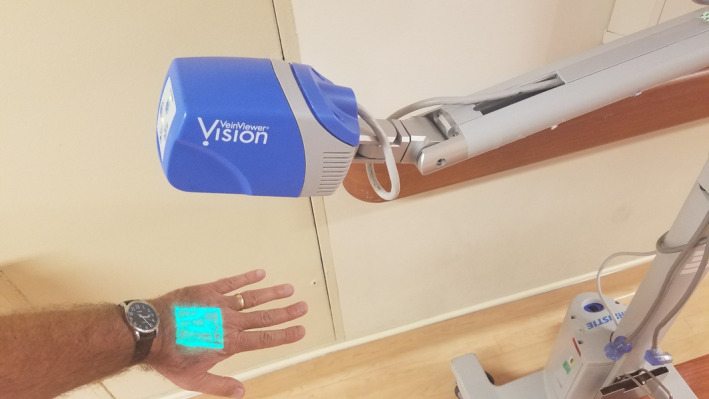
Vein finder

**FIGURE 2:**
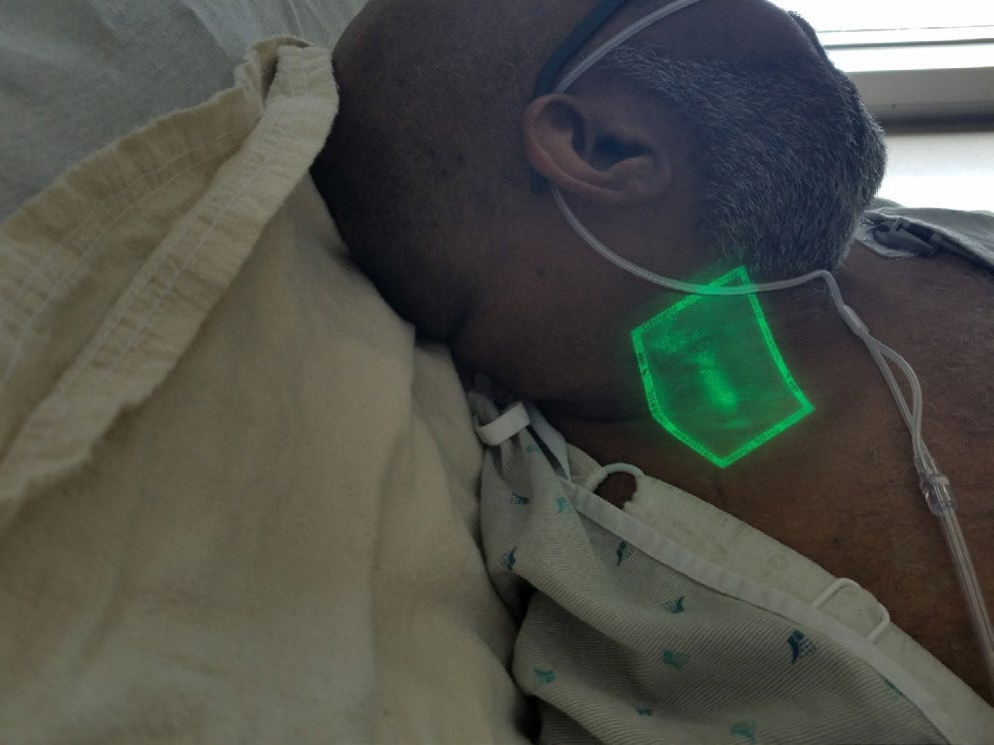
Vein finder enhancement of jugular venous distention

A 44‐year‐old man with a history of prior myocardial infarction, hypertension, and chronic kidney disease presented with a two‐week history of progressive dyspnea on exertion and orthopnea. Physical findings were limited by his body habitus, yet consistent with congestive heart failure (CHF) including mild tachypnea, basilar rales, and trace pedal edema. No S3 gallop was appreciated. Assessment of jugular venous pressure (JVP) was challenging due to his short stature and obesity. Admission B‐type natriuretic peptide and chest X‐ray corroborated the clinical suspicion of CHF. We used a bedside vein finder (Figure 1) to enhance visualization of jugular venous distention and monitor subsequent changes in JVP in response to therapy (Figure 2). The utility of our novel approach has yet to be further studied, but it may be of particular value in settings where point‐of‐care ultrasound is not immediately available.

## 2 DISCUSSION

### 2.1 Bedside assessment of jugular venous pressure

A vein finder light (Figure 1) can be used to dramatically improve the visibility of the EJV even with challenging situations such as obesity or darkly pigmented skin. Such devices utilize near infrared light to illuminate superficial veins and are generally accessible in most hospitals where they are typically used to augment placement of peripheral IVs.^1^ The vein finder light is safe, but it is only able to penetrate the skin and thus only able to assess the external jugular vein, not the internal jugular.

Despite limited sensitivity (~50%), fair specificity (~75%), and significant interexaminer variability,^2^ evaluation for elevated JVP remains a time‐honored method to establish the bedside diagnosis of CHF. While Lewis^3^ initially described the assessment of the external jugular vein (EJV) in 1930, other authors advise using the internal jugular vein,^4^ noting potential variability of the anatomy or presence of sclerotic valves of EJV. Sankoff and Zidulka described a method to assess for sclerotic valves, yet noted the EJV could not be seen in one‐third of patients, often related to obesity.^5^

### 2.2 How to examine neck veins

**FIGURE 3:**
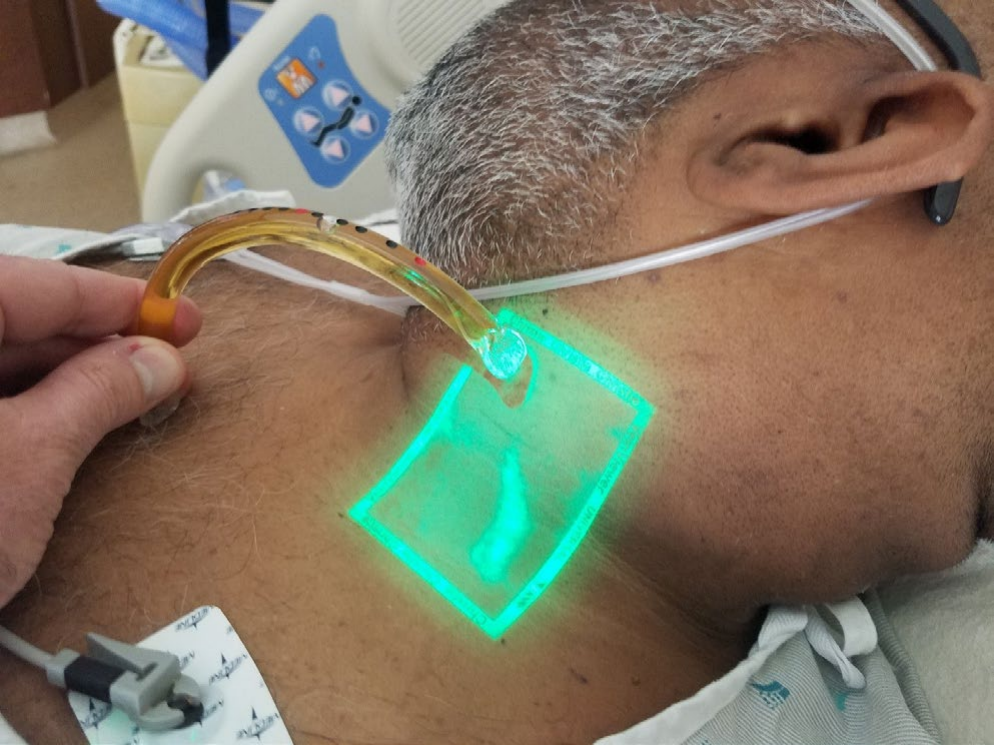
Venous arch used to measure jugular venous pressure (the venous arch can be purchased at https://www.daxtrio.nl/veneuze‐boog‐lewis‐borst_1.html)

The patient is positioned with head elevation between 30 and 60 degrees, allowing visibility of the venous meniscus of the jugular veins. The neck muscles should be relaxed by supporting the patient's head with a pillow, turning the head away and slightly elevating the jaw. To assess for potential EJV obstruction, the examiner should first occlude the EJV above the clavicle and observe for distention. Next, occlusion should be removed, observing for partial or full collapse. Complete collapse should indicate nonelevated central venous pressure (CVP, <8 cm of water). Incomplete collapse of the EJV may indicate elevated JVP, provided sclerotic valves or a fascial flap are not partially occluding the distal EJV. To assess for the latter, the EJV should next be occluded at the angle of the jaw and the vein milked downward with the examiner's other hand to cause vein emptying. Finally, the EJV should be observed for filling from below while the upper occlusion is maintained. The sternal angle is a convenient reference point assumed to lie 5 cm above the right atrium. A distended EJV then serves as manometer reflecting the pressure of the right atrium—that is, the vertical height of the EJV meniscus above the sternal angle when added to 5 cm approximates the CVP in cm of water. JVP values greater than 8 cm are considered elevated. Measurement of JVP can be facilitated using a plastic calibrated arched tube, a “venous arch.” This inexpensive “jugulometer” is a common component of physician's laboratory‐coat diagnostic equipment in the Netherlands^6^ (Figure 3).

### 2.3 Our patient

Our novel approach warrants further study, but enhanced our assessment of JVP. Initial JVP was elevated at 10 cm; admission B‐type natriuretic peptide and chest X‐ray corroborated our clinical suspicion of CHF.

## 3 CONCLUSIONS

Examining patient neck veins for the presence of JVD remains a valuable tool for establishing the bedside diagnosis of CHF. Visualization of neck veins is often difficult in patients with obesity or darkly pigmented skin. While designed to illuminate superficial veins and augment placement of peripheral IVs, vein viewer lights can be used to enhance visualization of the EJ and aid the estimation of JVP.

## CONFLICT OF INTEREST

The author has no conflicts of interest to declare.

## AUTHOR CONTRIBUTIONS

The corresponding author is the sole contributor to this presentation.

## ETHICAL APPROVAL

The patient has provided written consent the case to be published.
